# Therapeutic Hypothermia in Asphyxiated Neonates: Experience from Neonatal Intensive Care Unit of University Hospital of Marrakech

**DOI:** 10.1155/2017/3674140

**Published:** 2017-05-08

**Authors:** F. M. R. Maoulainine, M. Elbaz, S. Elfaiq, G. Boufrioua, F. Z. Elalouani, M. Barkane, Nadia El Idrissi Slitine

**Affiliations:** ^1^Neonatal Intensive Care Unit, Mohamed VI University Hospital, Marrakech, Morocco; ^2^Research Unit of Childhood Health and Development, Faculty of Medicine and Pharmacy, Cadi Ayyad University, Marrakech, Morocco

## Abstract

**Introduction:**

Therapeutic hypothermia (TH) is now recommended for the treatment neonates with hypoxic-ischemic encephalopathy (HIE). This treatment protocol is applied in our department since June 2012. The aim of this study is to report the first experience with head cooling in asphyxiated neonates in Morocco.

**Patients and Methods:**

Prospective study of newborns admitted for HIE from July 18, 2012, to May 15, 2014, in Neonatal Intensive Care Unit (NICU) of Mohamed VI University Hospital. The results were studied by comparing a newborn group who received hypothermia to a control group.

**Results:**

Seventy-two cases of neonates with perinatal asphyxia were admitted in the unit. According to inclusion criteria thirty-eight cases were eligible for the study. Only 19 cases have received the hypothermia protocol for different reason; the arrival beyond six hours of life was the main cause accounting for 41%. Complications of asphyxia were comparable in both groups with greater pulmonary hypertension recorded in the control group. The long-term follow-up of protocol group was normal in almost half of cases.

**Conclusion:**

Our first experience with the controlled TH supports its beneficial effect in newborns with HIE. This treatment must be available in all the centers involved in the neonatal care in Morocco.

## 1. Introduction

HIE in full term neonates is not unusual since it occurs among 1–3 newborns for 1000 live births [1]. The immediate and the long-term consequences can be serious. It is, indeed, responsible for a high mortality, globally, estimated at 23% of 4 million annual neonatal deaths [2, 3] and source of neurologic disability estimated at 25% according to the most recent meta-analysis [4]. These poor outcomes are virtually associated with the lack of any effective neuroprotective treatment following perinatal asphyxia, the management of which remained, until recently, supportive.

The incidence of HIE is significantly higher in developing countries this may present heavy social and economic costs. In Morocco perinatal asphyxia presents a large part of national public health policy; it is among the main causes of perinatal mortality but we do not have a published national epidemiological data. This high incidence is related mainly to socioeconomic factors: the lack of pregnancy follow-up, the lack of adequate infrastructure, the geographical distance of the delivery centers and consequently the persistence of home deliveries, the absence of structures adapted to the reception of the newborn at the delivery, and the lack of newborn transport policy.

TH as whole body or selective head cooling has become a standard therapy for moderate-severe HIE to reduce neurological damage. Most recent meta-analyses documented the efficacy of TH in term infants with moderate to severe encephalopathy [5, 6]. The safety of controlled hypothermia is now well established. No serious adverse events were reported to date by the randomized controlled studies [7]. Since the clinical benefits of TH are well established, it is considered the standard of care in many developed countries. In the United States, 50% of the neonatal intensive care units were reported to provide therapeutic hypothermia [8]. In Europe, therapeutic hypothermia is already implemented in several countries as well, due in part to participation of centers in clinical trials [9–12].

In Morocco, Mohamed VI University Hospital is the only center until recently (end of 2015), to implement TH for the treatment of asphyxiated neonates with HIE [13]. This study aims to assess the feasibility of using this protocol in this unit, to identify the problems encountered in its implementation, and to assess the outcome of these newborns.

## 2. Population and Methods

### 2.1. Population

This study was performed in the NICU at the Mohamed VI University Hospital of Marrakech, Morocco. This unit also receives children from all the south of Morocco. The hypothermia protocol was implemented in June 2012.

38 neonates out of 72 admitted for neonatal asphyxia in NICU from July 18, 2012, to May 15, 2014, were included in the study. These children were divided into two groups: the first group included 19 infants who were treated by hypothermia (protocol group) and a control group included 19 newborns with HIE but could not receive hypothermia.

### 2.2. Type of Study and Data Collection

This is a prospective study including neonates born in maternity of Mohamed VI Hospital (inborn) and neonates referred from other institutes (outborn). Data were collected from patients files, analyzing demographic parameters (gestational age, birth, and sex), perinatal-neonatal features (the origin of neonates, mode of delivery, acute intrapartum events, Apgar score at 1, 5, and 10 minutes, and the need of neonatal resuscitation), severity of HIE as assessed prior to cooling (Sarnat and Sarnat criteria), evolving information (hemorrhagic, infectious, renal complications, and death), and the result of the neurological examination at 6, 12, and 18 months. For infants in the protocol group we have registered the time of cooling initiation after birth, the rectal temperature monitoring, the adverse effects, and interventions during cooling.

### 2.3. Implementation of Hypothermia Protocol

All babies were selected and treated according to the local NICU protocol which was consistent with those recommended by French Society of Neonatology [14, 15], Briefly, newborn infants born at ≥36 weeks of gestation with birth weight > 1800 g were eligible for treatment if they had evidence of acute perinatal asphyxia and of moderate or severe HIE according to the Sarnat and Sarnat criteria. An amplitude-integrated electroencephalogram (aEEG) assessment for abnormal findings (as proposed by Al Naqeeb et al.) [16] prior to treatment initiation was highly supported. The exclusion criteria were severe intrauterine growth retardation with birth weight less than 1800 g, imperforate anus, head injury causing major intracranial hemorrhage, and severe chromosomal or congenital anomalies.

The selective cooling of the head was the hypothermia protocol employed in this work by using special apparatus (COOL CAP OLYMPIC). It should be started before the sixth hour of age. The objective of selective hypothermia was to reach a rectal temperature between 34 and 35°C for 72 h from the beginning of the procedure. At the admission, the newborn was installed in an infant warmer that was off unless the newborn had a temperature less than 34°C. Rectal temperature was checked every 15 min until obtaining a temperature of 34°C. Hypothermia was continued for 72 h while the rectal temperature was checked every 2 hours and the skin probe was held in place continuously. After 72 h of hypothermia, the newborn was gradually warmed from 0.2 to 0.4°C per hour (6 to 12 h). The newborn was under continuous monitoring with cardiopulmonary scope, a monitoring of diuresis, glucose monitoring every 6 to 8 hours, and monitoring of blood pressure every 2 hours (every hour during the warming). A close biological screening was carried out: daily chemistry panel, complete blood count, and coagulation profile.

### 2.4. Statistical Analysis

The results were expressed as number and percentage or by the average. Statistical analysis was carried out by SPSS 17. The difference between the two groups was studied either by the nonparametric Mann–Whitney test for quantitative variables or by Chi2 or Fisher test for qualitative variables. Statistical difference was considered significant if *P* < 0.05.

## 3. Results

After the implementation of the protocol in this unit, 38 neonates among 72 hospitalized for neonatal asphyxia had the indication of TH and were included in the study. The remaining 34 were excluded from the study because they did not meet the TH inclusion criteria.

The 38 neonates included in the study were divided into two groups; each one included 19 newborns: one group received hypothermia and the other was a control group.

There were various reasons for the 19 infants who were not cooled, mainly logistical: admission beyond 6 hours of life in 41%, the lack of place in the unit and the nonavailability of the cooling machine in 10%, technical problems in the machine in 15.7%, and cooling contraindication in 10% (extensive cephalohematoma, pulmonary arterial hypertension) and 20% because the diagnosis was not made early.

For the control group, we try to maintain hypothermia as long as possible, but it was difficult to maintain the temperature below 34°C for long time, so they were treated in normal temperature.

### 3.1. Population Characteristics

Maternal and neonatal characteristics (Tables 1 and 2) were similar in both groups. The neonatal asphyxia was suspected when fetal heart rate was abnormal (bradycardia and decelerations) in both groups. The Apgar score was lower than 5 at 5 min of life in all neonates from the control group and 17 in the protocol group. The population under study consisted of full term eutrophic newborns, mostly inborn. The HIE was classified as Sarnat 2 in half of the cases in both groups and showed severe electroencephalographic criteria (Table 2).

### 3.2. Protocol Assessment

Parents or caregivers of the asphyxiated neonates were informed about the important benefit of TH in the HIE. However, written consent was not mandatory for treatment initiation in this institution as cooling is considered a standard of care.

Newborns were admitted in the unit at 3.4 ± 4.6 h of life. The beginning of the procedure was at 3.3 ± 1 hour from early life. The average rectal temperature at admission was 33.4 ± 0.6°C. Among all the temperature readings, a rectal temperature below 33°C was recorded 10 times including 2 times in the same child; during this excessive hypothermia, the average time to stabilize the temperature over 33.8°C was 120 min.

### 3.3. Tolerance of Selective Hypothermia

Neonatal diseases associated with HIE were similar between the two groups except for pulmonary arterial hypertension that was paradoxically more common in the control group (Table 3). No statistically significant difference was observed for bleeding disorders. Bradycardia less than 90 beats per minute was noted in the protocol group which is considered a physiological effect of hypothermia. One newborn baby from the protocol group had presented a deformation of the head which is reported as side effect of head cooling therapy.

### 3.4. Evolution of Newborns

The mean duration of hospitalization was longer in the protocol group. Seven children died in the control group versus 3 in the protocol group (Table 4). Loss of views was more important in the control group 41% versus 15% in the protocol group; the follow-up rate at the age of 18 months was 81% in the protocol group versus 58% in the control group. 56% from the protocol group had normal neurological examination at 18 months of age, 20% presented different neurologic abnormalities (Table 5), and we recorded one case of death at the age of 16 months caused by status epilepticus.

## 4. Discussion

In this study, we present our experience with TH when preformed for the management of asphyxiated neonates with moderate and severe HIE, especially after the results of major randomized controlled studies that have shown a beneficial effect of controlled hypothermia on survival and long-term neurological outcome for newborns who suffered from HIE using either selective hypothermia [17, 18] or whole body cooling [10, 19–21]. Our results are encouraging with respect to the feasibility and safety of head cooling and the beneficial effect in terms of survival and neurodevelopmental outcome.

Two principal methods of cooling exist: selective head cooling and the total body cooling. No superiority of either modality is supported by the existing evidence [6, 22, 23]. However, the selective cooling is associated with a large gradient of intracerebral temperatures. The temperature difference measured at 2 cm depth of the cortical surface is typically 1.3 ± 1.1°C; it reached 7.5 ± 3.5°C during the selective cooling. The distribution of the intracerebral temperature is more homogeneous in the case of total body cooling (cortical area and deep brain gradient is 1.5 ± 1.2°C at baseline and 1.1 ± 0.9°C during the body cooling) [24, 25]. In this study, we used the selective head cooling for hypothermia.

The best neuroprotective effect is obtained if the treatment is started before 6 hours of life as shown in animal studies, where there is still a “therapeutic window” where secondary neuronal injury could be prevented or reduced by brain cooling [26, 27]. In fact it cooling babies before 3 h of age is even suggested by Thoresen et al. to obtain the optimal neuroprotective effect [28] and in the TOBY study, hypothermia was more effective in children treated during the first four hours after birth [20]. The average time for starting hypothermia was 3.3 ± 1 h in this study.

The need to start hypothermia before 6 hours of life has been a major limitation in this study since 41% of newborns referred in this center arrived too late, which explains the reason why the majority of cooled newborn neonates included in this study were inborn. This problem has already been discussed in other studies analyzing the feasibility of hypothermia in low- and middle-income countries [29] in which the therapeutic time-window for administering beneficial cooling may be already passed, due to delayed hospital admissions, prolonged or obstructed labor, and lack of neonatal transport facilities. It therefore seems necessary to improve the diffusion of this protocol within the perinatal network by promoting rapid transfer policy to level III centers of newborns with HIE.

Starting the hypothermia protocol before 6 h of life assumes a rapid assessment of the severity of HIE and therefore early recognition of anoxic-ischemic nature. Close and repetitive clinical assessment (every 1-2 h during the first 6 h) is required for these patients to determine the stage of HIE and if the HIE stage progresses from stage I to stage II therapeutic hypothermia should be started immediately. The recommendations of neonatal societies were made to this effect [14, 15], to guide practitioners in better recognition of the diagnosis of HIE and its severity. However, detailed neurological examination of an asphyxiated newborn according to criteria defined by Sarnat and Sarnat, originally during the first 48 hours of life [30], may be defective in some urgent assessment circumstances such as painful or sedated newborn. Olsen et al. suggest a neurological assessment repeated every hour in newborns with perinatal asphyxia and starting cooling if the patient develops any 3 out of 6 neurological findings [31]. The classical EEG or aEEG not only can confirm the severity of HIE and monitor subclinical seizures, but also redirect diagnostics. The combination of clinical and electrophysiological criteria seems the most efficient method to confirm encephalopathy (94% specificity, 85% positive predictive value, and 92% negative predictive value) [32]. In our unit a written protocol is used by the practitioner in call explaining clearly the diagnosis criteria, yet 20% of newborns were misdiagnosed because the clinical picture was not straightforward from early on.

Careful clinical and laboratory test is essential in newborns with HIE regardless of the mode of treatment, but TH requires additional parameters to be monitored throughout treatment such as umbilical arterial and venous catheterization for blood draw and urinary catheterization for urine output measurements. Full monitoring including heart rate, respiratory rate, blood pressure, core temperature, and SaO_2_ is required. The core temperature is recorded by esophageal or rectal probe. The axillary temperature measurements have been reported to give variable data and, therefore, should not be preferred over core temperature measurement methods [33]. The continuous monitoring of core temperature in this study was from a rectal probe.

The head cooling protocol requires specialized equipment; the hypothermia is obtained by using a special cap with circulating cold water placed on the head of the neonate. All the newborns from the protocol group were already hypothermic on admission with an average rectal temperature of 33.4 ± 0.6°C. We report difficulties maintaining the temperature between 34 and 35°C trying not to exceed the thermal objective of 35°C. Several episodes of hypothermia below 33°C were recorded twice in 8 newborns, reaching sometimes up to 32°C; this required sometimes the hanging of hypothermia by removing the cooling cap; it took almost two hours of the attending medical team to reach a temperature of 34°C. In the most of times the desirable temperature is maintained manually by changing the cap temperature or the environmental temperature. Achieving and maintaining hypothermia required the nurses constant attention and vigilance to ensure that the temperature remained within the prescribed range. This was the main obstacle in the TH; since it has involved a significant work charge to the medical and paramedical team whose number is insufficient. This lack of human resources could divert from taking care of other babies with a better prognosis.

The core temperature monitoring should continue for several hours after normothermia to avoid overshooting the rewarming [34].

Side effects related to perinatal asphyxia were similar for both groups; we observed a low rate in pulmonary arterial hypertension in the protocol group compared to the control group which is contradicting with the literature data [35]. In both groups, there was no difference in renal injuries; although the study of Róka et al. [36] suggested a beneficial effect of hypothermia in other organs such as the kidney and liver by reducing the cell lysis secondary to the anoxic-ischemic attack, this renal protective effect was even found in the work published by Delnard et al. [37] where they noted a significant decrease in transient renal failure rates in treated children.

Hospital stay was longer in the protocol group which was also found in the randomized study of Shankaran et al. [19]. This could be explained by the occurrence of neonatal infection. The worsening of neonatal infection is a major concern of cooling therapy in low- and middle-income countries related to an extensive literature on the association of increased mortality with hypothermia, and a potential worsening of sepsis with cooling, however, was reported [29, 38]. This fact could not be definitely concluded in the meta-analysis on the safety and efficacy of cooling therapy in low- and middle-income countries [39].

Almost all newborns diagnosed with HIE are started on empirical antibiotic treatment if the etiology of asphyxia is not clear. Hence, the hypothermia is known to cause some degree of immunosuppression with decreased leukocyte number and impaired functions [40, 41], as also reported in the latest Cochrane meta-analysis [7].

We had a higher rate of newborn follow-up in the cooling group comparing to a control group, reaching 80%. In the noncooling group, we had a high rate of loss of views; this may be explained by a possible improvement in their status. A significant decrease in death rates and neurological morbidity at the age of 18 months in children who have moderate or severe HIE was found in newborns of protocol group.

The limit of the study is the small number of included neonates. However, data presented here are derived from a single center and, therefore, only a limited number of neonates could have been assessed in a relative short time, particularly with respect to long-term outcome. On the other hand, the analysis of the results permits the detection of important clinical parameters which could allow further improvement in the clinical implementation of this novel therapeutic approach. The further studies with a larger population as well as training medical team are necessary to confirm these results.

## 5. Conclusion

Implementation of TH is facing a lot of problems in Morocco. The generalization of this practice needs to be guided by standardized protocols. Local protocols should be developed based on the existing international experience adapted to local context. Developing training programs, improving infrastructure including neonatal transport, and affording human resources are mandatory to guarantee the success of hypothermia in Morocco.

## Figures and Tables

**Table 1:** Maternal characteristics.

Maternal characteristics	Protocol group (*n* = 19)	Control group (*n* = 19)	*P* value
Maternal age (average)	26,3	27,3	NS
Parity (average)	1,3	2,1	NS
*Pregnancy complications, n* (%)			NS
Gestational diabetes	0	1 (5)	
Gestational hypertension	1 (5,2)	0	
*Delivery complications*			NS
Abnormal fetal heart rate	13 (68,4)	11 (57)	
Meconium or stained amniotic fluid	9 (47,4)	14 (73)	
*Peripartum pathologies*			NS
Prolapsed cord	2 (10,6)	2 (10,6)	
Other cordonal pathologies	0	1 (5)	
Retention of the after-coming head	2 (10,6)	0	
Shoulder dystocia	1 (5)	1 (5)	

NS: not significant.

**Table 2:** Neonatal characteristics.

Neonatal characteristics	Protocol group (*n* = 19)	Control group (*n* = 19)	*P* value
Female gender, *n* (%)	6 (31,6)	7 (36)	NS
Birth weight (g) (average)	3336	3300	NS
Apgar score ≤ 5 at 5 min, *n* (%)	17 (89,5)	19 (100)	NS
Apgar score ≤ 5 at 10 min, *n* (%)	12 (63,1)	16 (84)	NS
Intubation in the delivery room			NS
Intubation only	2 (10,6)	4 (21)	
Intubation and chest compression	3 (15,8)	2 (10,5)	
Features of aEEG, *n* (%)			NS
Type 2			
(i) With seizure	9 (47,3)	6 (32)	
(ii) Without seizure	1 (5,3)	2 (10,5)	
Type 3			
(i) With seizure	7 (36,8)	4 (21)	
(ii) Without seizure	0	2 (10,5)	
Electric seizure	2 (10,6%)	3 (15,8)	

aEEG: amplitude-integrated electroencephalography (according to Al Naqeeb classification).

**Table 3:** Complications during hospitalization.

	Protocol group (*n* = 19) %	Control group (*n* = 19) %	*P* value
Bradycardia <90/min	17 (90)	2 (10)	S
Thrombocytopenia	5 (26,3)	7 (37)	NS
Hepatic cytolysis	7 (36,8)	11 (57)	NS
Acidosis	4 (21)	7 (37)	NS
Hyperkalemia	7 (36,8)	3 (15)	NS
Renal failure	8 (42)	13 (68)	NS
PAH	0	6 (31)	S
Head edema	1	0	—
Nosocomial infection	5 (26,3)	4 (21)	NS

S: significant.

PAH: pulmonary arterial hypertension.

**Table 4:** Evolution during hospitalization.

	Protocol group *n* (%)	Control group *n* (%)
Average of hospital stay	12,4	6,7
Death	3 (15)	7 (37)
Normal neurological exam at discharge	8 (42)	5(26)

**Table 5:** Data from the neurological assessment at the age of 18 months.

	Protocol group *n* (%)	Control group *n* (%)
Normal neurological exam	9 (56)	5 (41)
Psychomotor delay	3 (15,7)	2 (10,5)
Epilepsy	1 (5,2)	1 (5,2)
Neurosensorial disorders	1 (5,2)	3 (25)
